# Preventing Maternal Deaths: Lessons Learned From the UK Confidential Enquiries Into Maternal Deaths and Morbidity

**DOI:** 10.1111/jog.70556

**Published:** 2026-11-09

**Authors:** Allison Felker, Marian Knight

**Affiliations:** ^1^ National Perinatal Epidemiology Unit, Nuffield Department of Women's and Reproductive Health University of Oxford Oxford UK

**Keywords:** health services, maternal mortality, obstetrics, public health surveillance, quality of health care

## Abstract

**Introduction:**

For more than seven decades, the United Kingdom (UK) Confidential Enquiries into Maternal Deaths (CEMD) have reviewed maternal deaths to identify lessons and improve care. This article summarizes key lessons from the enquiries, highlighting recent findings and enduring recommendations.

**Methods:**

The MBRRACE‐UK collaboration, which has delivered the CEMD program since 2012, collects information on all maternal deaths during pregnancy or up to 1 year after pregnancy in the UK and Ireland. Demographic and clinical surveillance data are collected to monitor trends, while medical records, postmortem reports, and relevant local investigations are anonymized and reviewed by multidisciplinary experts to identify key themes and opportunities for improvement.

**Results:**

Recent MBRRACE‐UK findings show an increasing trend in UK maternal mortality rates, which is predominantly driven by direct causes of deaths alongside persistent inequalities among women living in deprived areas and from some ethnic minority backgrounds. Confidential enquiries consistently emphasize the importance of individualized care, which considers women's circumstances including medical comorbidities, mental health, and social complexities. Improving outcomes requires joined‐up, multidisciplinary care extending beyond maternity services. Care before and after pregnancy is equally important to optimize outcomes.

**Conclusions:**

Despite consistent and recurring messages across confidential enquiries, maternal mortality in the UK is not declining. Sustained action is needed to ensure that lessons from maternal deaths are translated into meaningful and lasting changes in clinical practice, service delivery, and policy to reduce preventable deaths and improve maternal outcomes.

## 1 Introduction

A maternal death is a devastating event that carries profound and lasting impacts on the families left behind as well as those involved in the woman's care. For more than seven decades, the United Kingdom (UK) has sought to ensure that each loss leads to meaningful learning and lasting improvements in care through a rigorous confidential review process. Beginning in England and Wales in the 1950s, the UK's Confidential Enquiries into Maternal Deaths (CEMD) has evolved to encompass all four UK nations and now includes a topic‐specific, annual confidential enquiry into maternal morbidity. Since 2012, the MBRRACE‐UK (Mothers and Babies: Reducing Risk through Audit and Confidential Enquiries across the UK) collaboration has been responsible for delivery of the CEMD program in which multidisciplinary experts review every death during pregnancy or up to 1 year after pregnancy to identify causes, inequalities and modifiable factors contributing to maternal deaths. Grounded in confidentiality to encourage honest reflection, the enquiries produce actionable, practical recommendations aimed at improving future care through changes in clinical practice, public health policy, and political decision‐making. This article summarizes the lessons learned from the confidential enquiries including both recent findings and earlier recommendations that must not be forgotten.

## 2 Methods

The MBRRACE‐UK collaboration collects information on all maternal deaths in the UK and Ireland. Maternal deaths are defined as per the World Health Organization (WHO) criteria [1] and include any women who die during pregnancy or up to 1 year after pregnancy, from any cause, regardless of the length of the pregnancy or the pregnancy outcome. All deaths reported to MBRRACE‐UK are cross‐checked against national death and birth registries to ensure completeness and accuracy. For all women who die, MBRRACE‐UK requests the full medical records, postmortem reports and, where relevant, copies of any local reviews or investigations. All documents are fully anonymized prior to undergoing confidential review. Surveillance data including demographic and clinical information relating to the woman, her pregnancy and her birth are also collected for each woman to monitor trends in maternal characteristics. Each woman's care is examined by multidisciplinary expert reviewers and assessed against national guidance and standards. Subsequently, the expert reviews are examined by multidisciplinary chapter writing groups to enable the main themes for learning to be drawn out for the report.

## 3 Results

### 3.1 Maternal Mortality Trends in the UK

By global standards, maternal deaths are relatively uncommon in the UK. In 1952–1954, the first reporting period for the CEMD, the maternal mortality rate for England and Wales was 66 per 100 000 maternities, having fallen dramatically since the 1930s due to the widespread use of aseptic precautions and antibiotics to treat puerperal sepsis, and the introduction of blood transfusion and ergometrine for the treatment of postpartum hemorrhage [2]. At the time of the first MBRRACE‐UK report, published in 2014 and covering the years 2009–2011, this rate had again fallen significantly to 10.6 per 100 000 maternities (Figure 1) [3]. This decreasing trend was sustained until 2018–2020, when maternal mortality rates rose to 10.9 per 100 000 maternities [4]. Importantly, this triennium was the first to include the COVID‐19 pandemic; however, deaths associated with COVID‐19 infection cannot fully account for the rising rates. In the most recent reporting period of 2022–2024, the maternal mortality rate in the UK was 12.8 per 100 000 maternities or 12.5 per 100 000 maternities when deaths due to COVID‐19 were excluded [5]. While still substantially lower than the global maternal mortality ratio of 197 per 100 000 live births published in 2023 [6], this increase is a reminder of the continued importance of maternal mortality surveillance and review.

**FIGURE 1:**
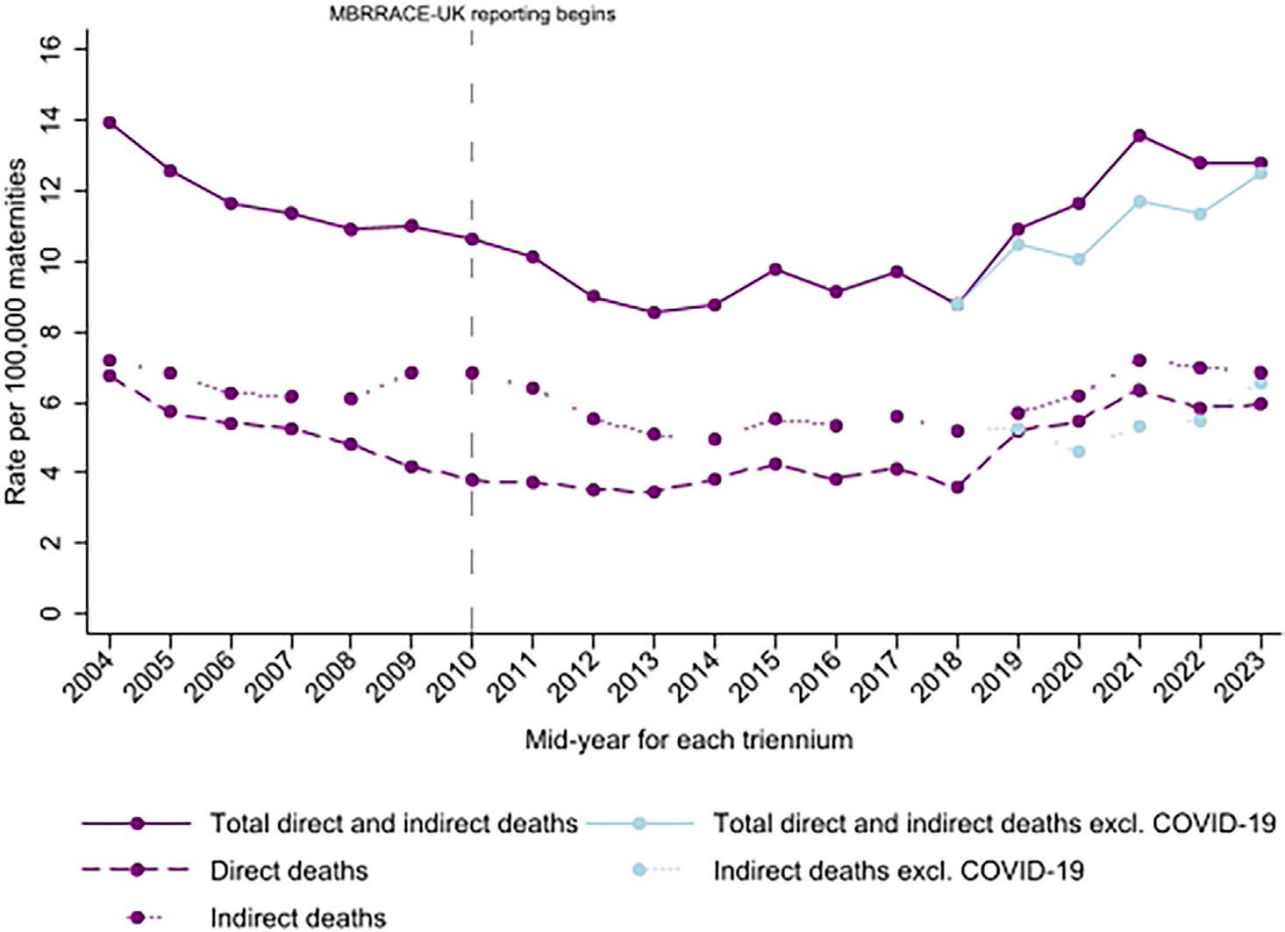
Three‐year rolling average maternal mortality rates per 100 000 maternities, UK 2003–2024.

In the 1950s, when the CEMD was established, the majority of maternal deaths were due to direct causes, or those associated with pregnancy or birth. In 1952–1954 there were 1094 direct maternal deaths, primarily due to preeclampsia, hemorrhage and pulmonary embolism [2]. Comparing this to 2022–2024, when there were 117 direct maternal deaths in the UK, it is clear that significant progress has been made to reduce what are largely thought to be preventable deaths. However, this progress has stalled in recent years, as the overall increase in maternal mortality rates is largely attributable to rising rates of direct maternal deaths (Figure 1). In 2020–2022 thrombosis and thromboembolism emerged as the leading cause of maternal death, occurring at more than twice the rate of any other direct cause of death [7]. As shown in Figure 2, the rate of maternal death due to thrombosis nearly doubled from 2009–2011 to 2021–2023. While some of the reasons behind this increase are evident in the lessons for care outlined later in this article, this rise is also reflective of the overall health of the current maternity population. The average maternal age in the UK has risen from 29.3 in 2008 to a record high of 30.9 in 2022 [8]. Similarly, in 2024, 27% of pregnant women were classified as obese [9] (the body mass index [BMI] ≥ 30 kg/m^2^) compared with 21% in 2015–2016 [10]. This is reflected in the characteristics of the women who died in 2022–2024; 40% of the women were over age 35 and 33% were obese [5].

**FIGURE 2:**
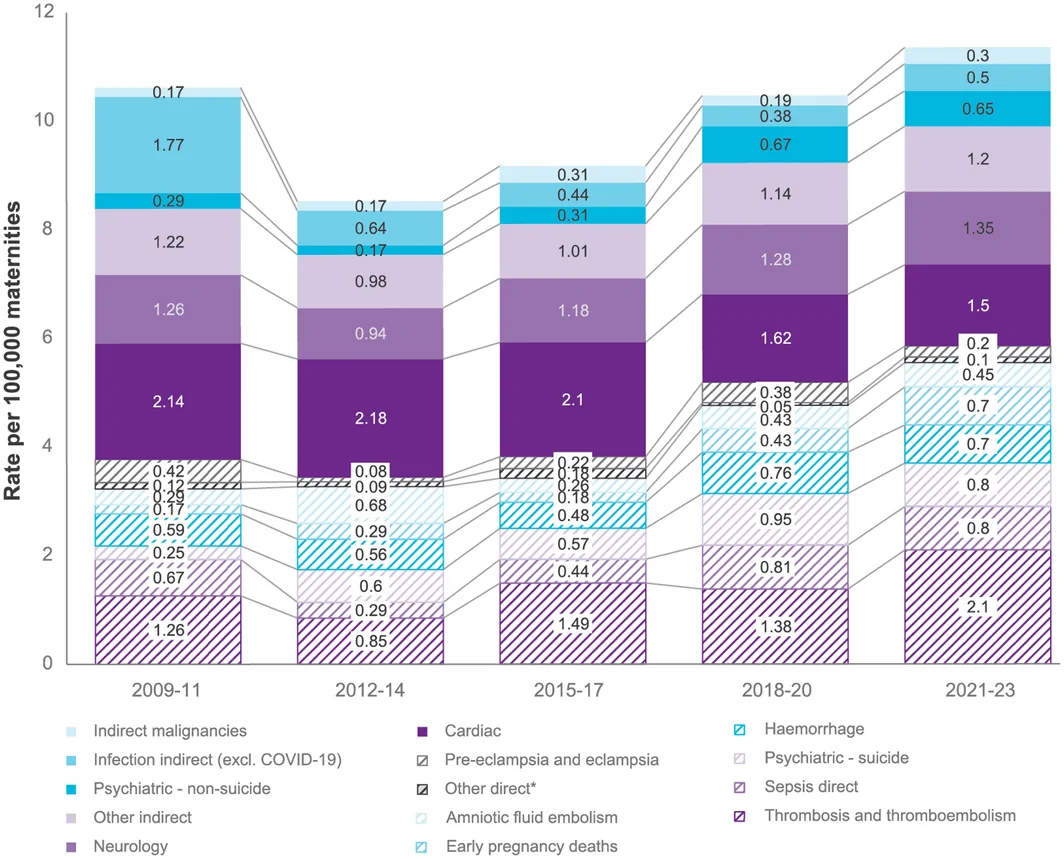
Maternal mortality rates by cause per 100 000 maternities, UK 2009–2023.

After thrombosis and thromboembolism, the next leading cause of direct maternal death in the UK is suicide, with rates increasing considerably since MBRRACE‐UK reporting began (Figure 2). Suicide was recognized as a direct cause of maternal death by the WHO in 2012 [1], highlighting the intersectionality of pregnancy and mental health. In 2021–2023, nearly half of the women who died had mental health disorders [11]. Many of these women also struggled with substance use or had other complex social risk factors, such as experiences of domestic abuse or social service involvement, which can compound mental health issues.

In contrast to direct maternal deaths, rates of indirect maternal deaths, due to preexisting or new conditions that may be aggravated by pregnancy, have remained relatively steady across the 12 years of MBRRACE‐UK reporting (Figure 1). The specific causes of indirect maternal death have also been fairly consistent, with deaths due to cardiac disease predominating in almost all triennia (Figure 2), with the exception of 2019–2020 and 2020–2022, when deaths attributable to COVID‐19 were the leading cause of indirect maternal death [7, 12]. Deaths due to cardiac disease have been a consistent focus of CEMDs, though the underlying causes have changed. In the 1950s, valvular disease due to rheumatic fever was the primary cause of cardiac death [2]; today ischemic heart disease and cardiomyopathy account for half of all maternal cardiac deaths [11]. As with deaths due to thrombosis and thromboembolism, this is likely reflective of changes in the general health of the maternity population and represents a potentially modifiable cause of maternal death.

### 3.2 Clinical Lessons for Care Learned From the Confidential Enquiries

#### 3.2.1 Prepregnancy Counseling

Many women who die have numerous preexisting medical comorbidities or risk factors that should be considered before becoming pregnant. However, as demonstrated in several confidential enquiries, many women with significant disease did not receive contraceptive advice or prepregnancy counseling regarding the risk to their health and life that pregnancy could pose. For instance, in 2020–2022, 21% of the women who died from cancer had been diagnosed and treated prior to becoming pregnant; many of these women did not seem to be aware that they could get pregnant [7]. Certain medical conditions can be significantly aggravated by the physiological changes of pregnancy. It is important that women are appropriately counseled on their risks and provided with effective contraception options. Similarly, the majority of the women who died from epilepsy had uncontrolled disease prior to pregnancy, but very few had received prepregnancy counseling or contraceptive advice [5, 12]. For women requiring ongoing medications, including those for epilepsy or hypertension, prepregnancy counseling offers an opportunity to adjust and optimize medications before planning a pregnancy and to make a plan for ongoing monitoring and management once a woman becomes pregnant.

Prepregnancy counseling is also important for women who may not have preexisting conditions but who do have significant risk factors. For instance, while only 11% of the women who died from cardiac disease in 2021–2023 had preexisting cardiac problems, many more had significant risk factors for cardiac disease including obesity and smoking [11]. This was also the case for many women who died from thrombosis and thromboembolism in 2020–2022. Of these women, 21% had a history of thrombosis, among other risk factors, and 25% died while in their first trimester of pregnancy. This means that many women died before they could be assessed for their risk of thrombosis or provided with thromboprophylaxis [7]. As for many others, these women would have benefited from prepregnancy counseling to discuss risk‐reduction strategies, such as weight loss or smoking cessation, and to help inform them of potential future risks and how to best address these in ensuing pregnancies, such as education on the importance of thromboprophylaxis.

#### 3.2.2 Prehospital and Emergency Care

Multiple confidential enquiries have demonstrated persistent variability in prehospital care for pregnant women including within the composition of the prehospital team, the level of training and the resources available. This variation can greatly affect decision‐making around on‐scene management including interventions such as resuscitative hysterotomy, which can help resuscitation efforts, or the provision of thrombolytic drugs for suspected pulmonary embolism [7] or magnesium sulfate for eclampsia [11]. Consistency across ambulance services and a clear framework to guide decision‐making can help reduce delays in transfer to hospital for definitive management. For many women, prolonged time on scene and delayed transfer to hospital impacted their outcome. This was especially true when the cause of the woman's collapse was not obviously apparent or if women were not known to be pregnant at the time of collapse, as was sometimes the case for women who had intra‐abdominal bleeding due to an ectopic pregnancy [7]. When women are conveyed to hospital, the enquiries have emphasized the importance of a prealert to the emergency department so that a multidisciplinary team, including an obstetrician and anesthetist, is available to assist.

#### 3.2.3 Joined‐Up Care and Senior Review

The majority of the women who died had multiple, interrelated medical, surgical, mental health and social care needs, which required coordinated, multidisciplinary care during pregnancy. It is important that women with complex or high‐risk conditions receive early triage for senior or specialist consultation so they can receive appropriate management without delays [11]. Joint, co‐located care facilities provide a coordinated approach to care that reduces the appointment burden for women and improves communication between teams. In the absence of joined‐up care pathways, fragmented care resulted in communication errors, limited information sharing and ineffective referral pathways for senior or specialist reviews. Often this resulted in inappropriate or delayed management of preexisting conditions, such as provision of antihypertensive medications [11] or adjustments to antiepileptic drugs [5, 12], missed recognition of uncommon symptoms, or delays in the provision of thromboprophylaxis for women with significant risk factors [7]. The provision of holistic, individualized care shared across disciplines is crucial to prevent complex women from falling through the gaps.

The importance of integrated, multidisciplinary care and senior review has also been noted in many acute care emergencies detailed in the confidential enquiries. For example, many of the women who died following an obstetric hemorrhage would have benefited from improved senior overview and situational awareness. There were several instances where the significance of their blood loss was underappreciated and the course of care was not appropriate [5, 12]. In other instances, limited planning meant that high‐risk cesarean sections were sometimes conducted without senior availability from anesthetics, urology, general surgery or interventional radiology, which limited the ability to respond to emergencies [5, 12]. Forward planning with multidisciplinary input can help guide management and improve responses in such situations.

#### 3.2.4 Recognition of Symptoms

The confidential enquiries have shown consistent evidence of normalization bias or attribution of women's symptomology to pregnancy or a simple self‐limiting condition without proper investigation and diagnosis. This was particularly apparent during the COVID‐19 pandemic, when many women's symptoms, including shortness of breath, fever, nausea and chest pain, were attributed to COVID‐19. Notably, this occurred among women who died from other infectious causes who had similar symptomology but whose misdiagnosis resulted in delayed recognition of sepsis and inappropriate treatment methods [12]. Similarly, symptoms of nausea or vomiting were often attributed to pregnancy without consideration of other associated symptoms or the time of onset. In some instances, clear symptoms of uncommon conditions were missed since maternity staff were unfamiliar with them, as was the case for women with Addison's disease who presented with changes in skin tone alongside vomiting and weight loss [12]. In other instances, staff outside maternity services may not have considered pregnancy as a possibility as was the case for many of the women who died from ectopic pregnancy who presented with atypical symptoms but did not have a pregnancy test to exclude pregnancy‐related causes [7]. In all instances when a woman presents with abnormal, unexplained symptoms it is important to obtain a full clinical history and conduct a differential diagnosis to distinguish between conditions with similar symptoms. It is not enough to exclude a diagnosis; a diagnosis must be made so that the woman can be appropriately treated whatever the underlying cause.

“Red flag” signs or symptoms have been highlighted across many confidential enquiries. This includes certain headache symptomology such as sudden‐onset, “worst ever” headaches, headaches that persist or have associated symptoms or headaches requiring opioids [12], as well as particular symptoms in women who present with chest pain or breathlessness [11], which may indicate underlying heart disease. When women present with “red flag” symptoms, it is important to consider the whole picture including a woman's individual risk factors, such as elevated blood pressure, and their personal and family history, which may help guide follow‐up investigations.

“Red flag” symptoms are not always physical. For women with mental health issues, where decline can be rapid, it is important to conduct an urgent assessment if they present with a recent significant change in their mental state, including the emergence of new symptoms, new thoughts or acts of self‐harm, or expressions of incompetency as a mother or estrangement from their child [11].

Often women reviewed in the confidential enquiries presented on multiple occasions with the same symptoms. Pregnant and recently pregnant women are generally young and healthy and will not present to the emergency department or their primary care physician unless they are feeling very unwell. This is particularly true of postnatal women who have young babies at home. As such, when women have repeat presentations, especially in a short period of time, there is a need for inquisitiveness. It is also important to consider that the repeat presentations themselves may be symptomatic of larger issues beyond physical health. For instance, one indicator of possible domestic abuse is repeated health consultations with no clear diagnosis. Other indicators include chronic, unexplained pain or gastrointestinal, genitourinary or gynecological symptoms [11]. In all instances it is important to recognize the pattern of attendance and escalate concern, which may necessitate senior or specialist review or follow‐up investigations.

To help identify potential deterioration and a need for escalation, the confidential enquiries have repeatedly emphasized the use of maternity early warning scores (MEWS) for all pregnant or recently pregnant women, regardless of their location of care [5]. Typical early warning scores used in routine care are not well validated in obstetric populations and do not correlate with obstetric physiology [4]. As such, the use of these tools in place of MEWS may underestimate women's symptoms and result in delayed recognition of deterioration.

#### 3.2.5 Postnatal Care

To align with global figures, UK maternal mortality rates include only women who die during pregnancy or up to 6 weeks after pregnancy; however, mortality rates for women between 6 weeks and 1 year after the end of pregnancy have been steadily increasing. Between 2009–2011, when MBRRACE‐UK reporting began, and 2022–2024, the rate of late pregnancy‐associated maternal deaths has increased by 20%. The majority of deaths in this period are mental‐health related (33%) or associated with medical conditions such as cardiac disease [5]. Care for women with mental health conditions, particularly those who are vulnerable, often deteriorates postnatally when they are no longer regularly engaging with services and the focus of support services shifts to the care of the baby [11]. Fragmented postnatal care was also evident for many women with medical comorbidities, such as diabetes or epilepsy, who often had joined‐up, multidisciplinary, specialist care during pregnancy that disappeared after discharge from maternity services [5].

In order to ensure continuity of care postnatally, there must be proper communication and documentation at handover. For some women, there was delayed recognition of their acute deteriorating condition as postnatal visits did not include any observation or assessment of women's vital signs [5, 12]. For other women, there was limited recognition or appreciation of deterioration related to known medical conditions including uncontrolled blood pressure or increasing seizure frequency. It is vital that information about any medical, mental health or social conditions that require ongoing support and management is clearly documented in the discharge summary provided to primary care. Senior oversight at discharge from hospital can help identify these issues and develop a clear, comprehensive plan for postnatal care. This plan must include information on the management and monitoring of any required medications, including antihypertensive or antiepileptic drugs, which also specifies clinical targets, timeframes and responsibilities [11, 12].

This should apply to conditions preceding the index pregnancy as well as those newly developed during pregnancy. Risk is not static; it is imperative that women are reassessed postnatally as their risks may have changed. This is demonstrated by the care of women who died from thrombosis and thromboembolism in 2020–2022, many of whom were not reassessed postnatally meaning that changes to their risk profiles such as weight gain, pregnancy complications or an increased parity, were not recognized and managed appropriately [7]. It is also important that clinicians and women are aware of other pregnancy‐induced risk factors, such as gestational diabetes or preeclampsia, which may have long‐term health effects requiring follow‐up and intervention [11].

### 3.3 Messages for the Care of Women With Mental Health and Social Complexities

#### 3.3.1 Inequalities in Maternal Mortality

Although the CEMD has always considered the wider social circumstances of the women who died, the first specific enquiry into health inequalities was not published until 1980 [2]. Unfortunately, the findings of this report, including higher rates of death among women living in more impoverished areas, are largely unchanged in current MBRRACE‐UK reporting. As in the first report on health inequalities, rates of maternal death for women living in the most deprived areas were two times higher than for women living in the least deprived areas in 2022–2024 [5].

Similar, persistent inequalities are also observed in the rate of maternal death for women from Black and Asian ethnic backgrounds. In 2009–2011, the rate of maternal death was three times higher for Black women and two times higher for Asian women compared with White women [3]. These figures have changed little over the course of MBRRACE‐UK reporting. In 2021–2023, the rates of maternal death for Black and Asian women were 28.21 and 16.74 per 100 000 maternities, respectively, compared with 12.44 per 100 000 maternities for White women [5]. Differences in the rates of specific causes of maternal death were also observed in 2021–2023, with Black women dying from thrombosis and thromboembolism at nearly three times the rate of White women and Asian women with fourfold higher rates of maternal death due to COVID‐19 than White women [11]. However, inequalities in maternal mortality rates for Black and Asian women persist even after adjusting for medical comorbidities, smoking status and BMI [13, 14] suggesting that preexisting conditions or health behaviors alone cannot explain these differences in maternal mortality rates or causes of maternal death. Similarly, Black women remain at a higher risk of maternal death independent of social deprivation, with similar rates of death reported among Black women in all socioeconomic groups [14]. Addressing these disparities in maternal mortality and considering the intersectionality of medical, mental health and social complexities must remain an important focus to prevent maternal mortality.

#### 3.3.2 Recognition of Social Complexity

As first demonstrated in the 2020 MBRRACE‐UK and recently re‐emphasized in the 2025 report, almost all of the women who die during pregnancy or up to a year after pregnancy in the UK experience a “constellation of biases.” This includes deprivation and ethnicity as noted above, but also encompasses other complexities related to pregnancy, physical and mental health and other social circumstances that can impact how they engage with care and the care they receive [11, 15]. A consistent message across many confidential enquiries has been the need to recognize women's complex care needs through consistent assessment and recording of social risk factors. This includes ensuring that women are seen alone, without their partner or family member on at least one occasion so that sensitive inquiries can be made about domestic abuse [11]. It also must consider deprivation, financial hardship and digital poverty when requiring women to take time off work or travel for appointments or access vital health information using a smartphone [7, 11]. Due to the sensitive and often stigmatizing nature of many social risk factors, such as domestic abuse or substance use, it is important that these conversations are revisited more than once in pregnancy once healthcare providers have developed a trusting relationship with the women they care for [11].

#### 3.3.3 The Need for Coordinated Care

Many women with multiple complexities will often require input from various services and agencies outside of routine maternity care including mental health or substance use services or social care referrals. Another lesson learned from the confidential enquiries is to ensure that women with multiple adversities receive holistic, individualized care that recognizes and tries to ease the burden of appointments through interagency working and joined‐up, coordinated care. It is also important to properly safeguard women when they miss appointments or do not engage with care. Multiple missed appointments should be regarded as a potential “red flag.” Women should not be discharged from services after one missed appointment. Instead, this information should be shared between agencies and the reasons for non‐engagement explored with appropriate follow‐up or escalation to help women attend appointments [7, 11, 16]. Similar information sharing and interagency working is needed for women with known domestic abuse. Too often in the confidential enquiries, limited communication between healthcare providers and agencies meant that key information was not known to all those caring for the woman, sometimes resulting in adverse outcomes [11]. Women must be continuously supported throughout pregnancy and the postnatal period, especially when there are safeguarding concerns.

#### 3.3.4 Barriers to Access and Engagement

While many women will experience vulnerability, certain groups may face additional barriers in accessing care including ethnic minority women, women with social service involvement, or women with language barriers. Confidential enquiries into the care of recent migrant women found that most women did not have their language needs properly assessed or documented in their maternity records [7, 17, 18]. When women were found to have language difficulties, the provision of professional interpreter services was inconsistent, and important written information, including consent forms, was given in English, which may have impacted their ability to make informed decisions about their health [7, 17, 18]. Evidence of microaggressions was also evident for many of the migrant women included in the confidential enquiries, including inaccurate recording of ethnicity or nationality [7]. Similar examples of microaggressions and racism have been noted in confidential enquiries reviewing the care of Black and Asian women [13, 19, 20]. Other biases in care were also evident for both migrant and ethnic minority women, as well as women who had social service involvement before their death, including the use of stigmatizing language [4, 21]. Improved guidance and training are necessary to help clinicians recognize the broader social complexities of the maternity population and provide appropriate, trauma‐informed care.

#### 3.3.5 Mental Health Conditions

Mental‐health related conditions are among the most common causes of maternal deaths in the UK, especially in the period between 6 weeks and 1 year after the end of pregnancy when they account for over a third of all deaths [11]. Of all the women who died from mental‐health related causes in 2021–2023, a quarter were considered to have severe and multiple disadvantages including over half of the women who died from substance use [11]. Many of these women had a history of childhood trauma, including abuse or social care involvement, and many were living in unstable housing or had experienced domestic abuse as an adult [11]. Women with complex trauma histories can be particularly vulnerable to worsening mental health in the perinatal period, especially if they experience a loss event, including an early pregnancy loss, bereavement or custody loss of their baby or older children. These women require additional supports during and after pregnancy; however, it has been noted in several confidential enquiries that women who experience a loss are often excluded from specialist perinatal mental health services and any support for the mother provided by social services during child safeguarding procedures typically falls away after the child is removed from their care [11, 16]. Thus, in many instances, it is the most vulnerable women who are falling through the gaps in care.

### 3.4 Equity in the Care of Pregnant Women

Pregnant and breastfeeding women have long been excluded from research, including clinical trials for new treatments and vaccines. As such, there is often little evidence to guide clinical and medical management and many women do not receive appropriate imaging or treatment during pregnancy. For instance, many of the women who died from stroke had clear risk factors or symptoms including abnormally high blood pressure or severe headaches. However, further investigations, including MRI brain imaging, were not undertaken due to pregnancy [5, 12]. This was also the case for women who had new or recurrent cancer during their pregnancy, many of whom experienced delays in diagnosis and staging as clinicians were hesitant to conduct mammography or CTs. This was true for women with newly diagnosed cancer as well as those with previous cancer diagnoses where there was a high suspicion of recurrence [7]. A recurring message from the confidential enquiries is that most imaging modalities are safe during pregnancy and women should not be inappropriately denied them simply because they are pregnant.

This message is also true for condition‐specific treatments, such as chemotherapy, which, despite being safe in pregnancy, were often postponed until the postnatal period resulting in delayed management and exacerbated disease [7]. Similarly, highly variable advice on the provision of extracorporeal membrane oxygenation (ECMO) in pregnant women meant that many women with severe respiratory illness due to COVID‐19 infection were not considered for ECMO or were referred too late for them to benefit [12]. This is not a new phenomenon, as similar findings were observed in the review of the care of women who died from H1N1 influenza in 2009–2012 [3]. Some women with COVID‐19 were also denied medical treatments, including tocilizumab, because they were pregnant [12] and several women with H1N1 had delayed diagnosis and initiation of antiviral therapy [3]. It is imperative that pregnant women are provided with the same standard of care as the general population unless there is a clear reason not to.

The exclusion of pregnant and breastfeeding women from clinical trials has also resulted in widespread vaccine hesitancy among both clinicians and pregnant or postpartum women due to confused messaging and limited research evidence. Evidence from the recent confidential enquiries into maternal deaths associated with COVID‐19 suggests that vaccination was not discussed with or offered to most of the women who died [12, 22]. None of the women who died from COVID‐19 in 2019–2021, and who were eligible for the vaccine prior to their death, were fully vaccinated [12]. Similarly, while some women who died from COVID‐19 in 2022–2024 had been vaccinated prior to pregnancy, all declined booster doses in pregnancy and the majority (60%) remained completely unvaccinated [5]. This was also the case for the women who died from influenza in 2009–2012, the period encompassing the H1N1 pandemic, none of whom had received a seasonal flu vaccine or the H1N1‐specific vaccine prior to their death [3]. Since the H1N1 pandemic, influenza immunization rates remain low in pregnant women despite widespread guidance recommending their safe use in pregnancy; none of the four women who died from influenza in more recent enquiries was vaccinated [5, 12]. Public health messaging must continue to target immunization rates in pregnant women to prevent future deaths due to influenza, COVID‐19 or other pathogens yet to emerge. The inclusion of pregnant and breastfeeding women in research can help ensure that messaging is evidence‐based and unambiguous about the benefits and risks of vaccination.

## 4 Conclusions

Although maternity care in the UK has advanced significantly since the confidential enquiries were first introduced over seven decades ago, the reviews of the women who died continue to provide crucial lessons for improving care and preventing future deaths. Persistent inequalities in maternal mortality highlight the ongoing need to place women at the center of these reviews and to consider the full context of their lives. This includes medical comorbidities but also mental health problems and wider social circumstances that may influence access to and engagement with care. Addressing these complex and interrelated factors requires a holistic approach, supported by effective multidisciplinary collaboration and appropriate senior oversight. These lessons are not new, nor are they unique to the UK. A sustained commitment to learning from maternal deaths and implementing these findings in practice is essential to improve maternal outcomes.

## Author Contributions

**Allison Felker:** writing – original draft, writing – review and editing, data curation, conceptualization, visualization, formal analysis, project administration. **Marian Knight:** conceptualization, funding acquisition, writing – review and editing, project administration, supervision, resources.

## Funding

This work was supported by Healthcare Quality Improvement Partnership (NCA 2141) and National Institute for Health and Care Research (NIHR201333).

## Disclosure

The content of this article, including all data and confidential enquiry findings, is derived from reports previously published by the MBRRACE‐UK collaboration.

## Ethics Statement

The authors have nothing to report.

## Consent

Identifiable MBRRACE‐UK data in England and Wales was collected without consent with approval of the Secretary of State for Health and Social Care under Section 251 of the NHS Act 2006 (15/CAG/0119). Data from Scotland were collected by MBRRACE‐UK without consent with approval from the Public Benefit and Privacy Panel for Health and Social Care (PBPP 2223–0101). Identifiable information was not collected from Northern Ireland or the Republic of Ireland.

## Conflicts of Interest

A.F. and M.K. are part of the Maternal, Newborn and Infant Clinical Outcome Review Programme (MNI‐CORP), delivered by MBRRACE‐UK and commissioned by the Healthcare Quality Improvement Partnership (HQIP NCA 2141) on behalf of NHS England and the governments of Wales, Northern Ireland, Jersey, Guernsey, Scotland, and the Isle of Man as part of the National Clinical Audit and Patient Outcomes Programme. M.K. also is funded through an NIHR Senior Investigator Award (NIHR201333). No authors have other relationships, activities, or conflicts of interest that could appear to have influenced the submitted work.

## Data Availability

Data sharing is not applicable to this article as no datasets were generated or analyzed during the current study.
